# Self-reported medication use validated through record linkage to national prescribing data

**DOI:** 10.1016/j.jclinepi.2017.10.013

**Published:** 2018-02

**Authors:** Jonathan D. Hafferty, Archie I. Campbell, Lauren B. Navrady, Mark J. Adams, Donald MacIntyre, Stephen M. Lawrie, Kristin Nicodemus, David J. Porteous, Andrew M. McIntosh

**Affiliations:** a Division of Psychiatry, Royal Edinburgh Hospital, University of Edinburgh, Edinburgh EH10 5HF, UK; b Generation Scotland, Centre for Genomics and Experimental Medicine, Institute for Genetics and Molecular Medicine, Western General Hospital, University of Edinburgh, Edinburgh EH4 2XU, UK; c Institute for Genetics and Molecular Medicine, Western General Hospital, University of Edinburgh1, Edinburgh EH4 2XU, UK; d Centre for Cognitive Ageing and Cognitive Epidemiology, University of Edinburgh, 7 George Square, EH8 9JZ, UK

**Keywords:** Agreement, Pharmacoepidemiology, Self-report, Medicines, Indication, Linkage

## Abstract

**Objectives:**

Researchers need to be confident about the reliability of epidemiologic studies that quantify medication use through self-report. Some evidence suggests that psychiatric medications are systemically under-reported. Modern record linkage enables validation of self-report with national prescribing data as gold standard. Here, we investigated the validity of medication self-report for multiple medication types.

**Study Design and Setting:**

Participants in the Generation Scotland population-based cohort (*N* = 10,244) recruited 2009–2011 self-reported regular usage of several commonly prescribed medication classes. This was matched against Scottish NHS prescriptions data using 3- and 6-month fixed time windows. Potential predictors of discordant self-report, including general intelligence and psychological distress, were studied via multivariable logistic regression.

**Results:**

Antidepressants self-report showed very good agreement (κ = 0.85, [95% confidence interval (CI) 0.84–0.87]), comparable to antihypertensives (κ = 0.90 [CI 0.89–0.91]). Self-report of mood stabilizers showed moderate-poor agreement (κ = 0.42 [CI 0.33–0.50]). Relevant past medical history was the strongest predictor of self-report sensitivity, whereas general intelligence was not predictive.

**Conclusion:**

In this large population-based study, we found self-report validity varied among medication classes, with no simple relationship between psychiatric medication and under-reporting. History of indicated illness predicted more accurate self-report, for both psychiatric and nonpsychiatric medications. Although other patient-level factors influenced self-report for some medications, none predicted greater accuracy across all medications studied.

**What is new?**

**Key findings**
• Self-reported medication use shows high validity in the general population, although there is variation between medication classes. A simple relationship between psychiatric medications and under-reporting was not found. Antidepressant reporting agreement is comparable to other long-term nonpsychiatric medications.

**What this adds to what was known?**
• Medical history of an indicated health condition is the strongest predictor of accurate report. General intelligence was not associated with the accuracy of reporting.

**What is the implication and what should change now?**
• Medication-related factors such as range of indications, prescribing cycles, and phrasing of self-report question may also influence accuracy of self-report. Longer fixed time windows produce higher levels of agreement and positive predictive values, at the expense of some loss of sensitivity.

## 1 Introduction

Cohort studies, and other epidemiologic studies using self-reported data, depend on the accuracy of the self-report to make accurate and reliable conclusions. This includes pharmacoepidemiologic and large-scale biobanking studies which are based on self-reported medication use. Self-reported medication use can be determined by questionnaire [1], [2]; by telephone or internet survey [3]; or by face-to-face interview [4], [5], [6], [7]. However, self-report is subject to recall errors and biases [8], [9] and patients may be less willing to disclose details of certain medications than others.

The accuracy of self-report can be verified by comparison to a trusted measure or “gold standard.” For medication utilization, the choice of gold standard depends to an extent on the purpose of the study (i.e., estimating patient adherence or monitoring prescribing behavior of clinicians), and there is therefore no universally applicable and accepted gold standard [10], [11]. One option is for a third party to perform a home inventory [12] or record individual medications produced by the patient [13], but these assessments are difficult to perform on a large scale. An alternative is to compare self-report data with prescriptions, healthcare insurance claims, or general practice medical records [4], [5], [11], [14]. Prescribing databases have been shown to be highly accurate in recording medication utilization [15], at least for those medications that require prescriptions.

Among published studies comparing medication self-report to prescribing data, the majority have been relatively small in size [4], [6], [7], [10], [11], [12], [13], [16], [17], [18]. Many studies are restricted to certain medications or medication types, such as antihypertensives [11]; cardiovascular drugs [6]; antidepressants [17], or hormone replacement therapy (HRT) [1]; or to special populations, such as the elderly [6], [12], [15]; postmenopausal women [2], [5]; or psychiatric illnesses [16]. Few studies use large population-based samples [4], [13], [14], [19] or multiple disparate medication types [13], [19], [20], [21]. Such comparisons are important, however, for they enable study of systematic over- and under-reporting of medication utilization between drug classes.

Self-report can be compromised by a number of factors, including not understanding the question, poor recall, and intended nondisclosure [4]. There is no consensus on patient-level factors predisposing to discordance between medication self-report and gold standard measures, but previous reports have implicated advancing age [9], [19], being unmarried [19], [21], number of medications regularly dispensed [18], [22], suffering poor health [19], and lower educational attainment [21]. Within medication classes, there is some evidence that psychiatric medications are less likely to be accurately self-reported [19], [22]. Potential explanations for this include confusion regarding medication indication but also nondisclosure because of social desirability bias [9] or self-stigmatization [2], [4], [10], [23]. Factors that have not to date been found to influence reporting include gender [19], [21] and cognitive health [21].

Prescribing data can be sourced from local health providers or insurers [10], pharmacy records [6], [11], [13], [14], [17], [21], social insurance databases [16], [19] or national health service databases [1], [2], [4]. The recording of the dispensing and collection of medication, as well as its prescribing, is important for studies that seek to measure patient utilization (although even collection of a medication is not a hard indicator of usage). The country of origin of the study and respective prescription legislation, dispensing, and reimbursement practices are also relevant to interpreting self-report against prescribing data (e.g., over-the-counter medications may not appear in these data) and to make comparisons between national studies.

In this study, we sought to ascertain agreement between medication self-report, derived from a large UK cohort study, compared with record-linked national prescribing data as gold standard, across a range of commonly used psychiatric and nonpsychiatric medications. We hypothesized that agreement would be lower for psychiatric medication types because of systemic under-reporting. To our knowledge, this is one of the largest population-based studies of medication self-report, also incorporating a covariate analysis method across a range of medications.

## 2 Methods

### 2.1 Study population

Our study used the Generation Scotland: Scottish Family Health Study (GS:SFHS) family-based and population-based cohort of Scottish adult volunteers (*n* = 21,474), recruited February 2006 to March 2011, which has been described elsewhere [24], [25]. The cohort has a higher proportion of females (59%) and older median age (47 males: 48 females) than the Scottish population at the 2001 census (37 and 39, respectively) [25], [26]. Written informed consent was obtained for 98% of GS:SFHS for data linkage to routinely collected healthcare records.

### 2.2 Medication self-report data

All participants in GS:SFHS were asked to complete a pre-clinic questionnaire before their enrollment in the study. The first phase of the study used a text-based questionnaire which is not part of this analysis. Those individuals recruited between June 2009 and March 2011 (*n* = 10,980, 59.5% female) completed a coded questionnaire where the Medications section was a “Yes” vs. “No” checkbox, with the accompanying question “Are you regularly taking any of the following medications?” The available options were (1) “cholesterol-lowering medication (e.g., Simvastatin)”; (2) “blood pressure–lowering medication”; (3) “insulin”; (4) “hormone replacement therapy”; (5) “oral contraceptive pill or mini pill”; (6) “Aspirin”; (7) “antidepressants”; and (7) “mood stabilizers.” The completed questionnaires were then machine read and electronically recorded using anonymized patient linkers.

### 2.3 Additional covariate data

**Fig. 1:**
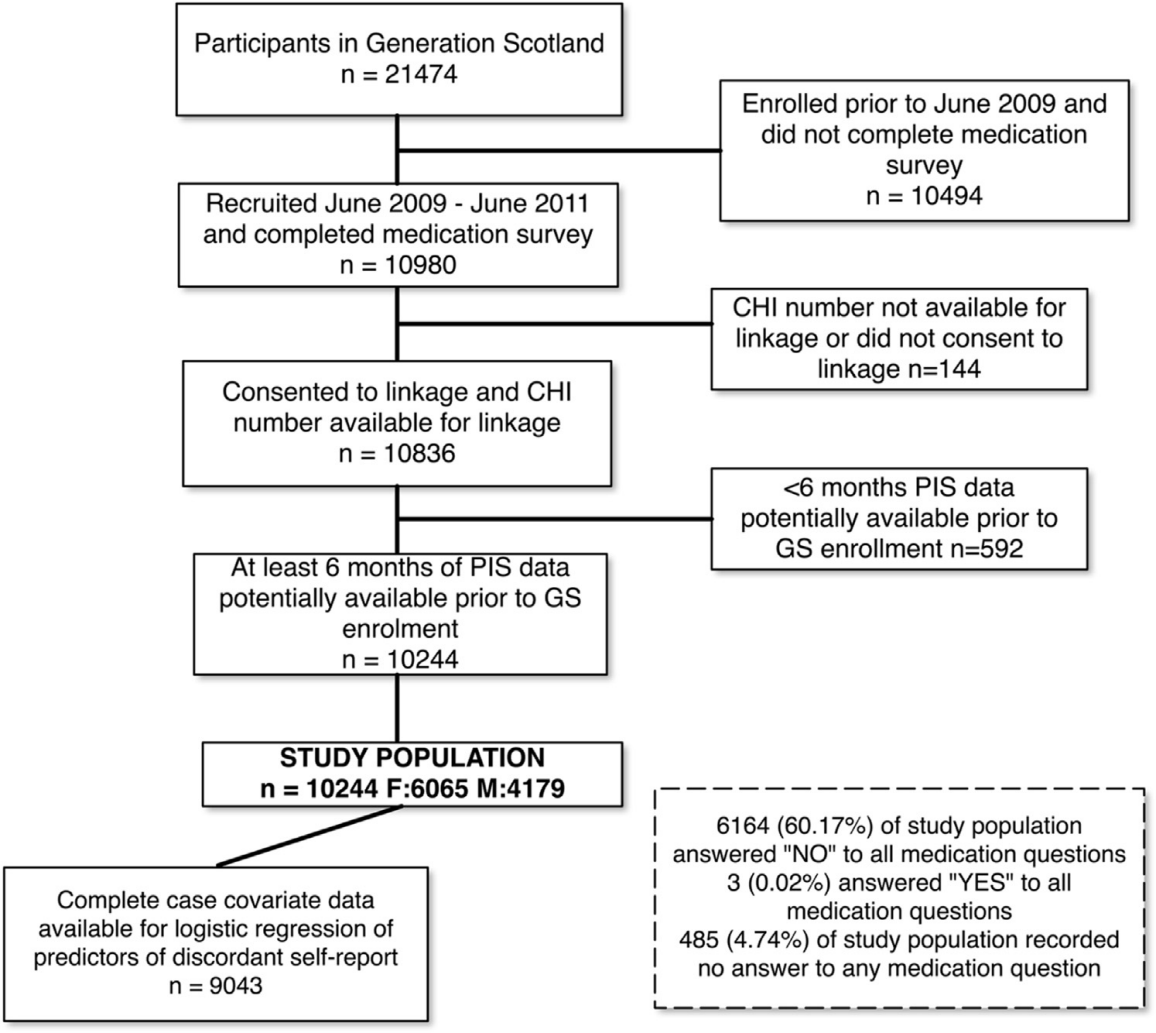
Flowchart of derivation of study population and subset used in logistic regression analysis, from the Generation Scotland cohort. CHI, Community Health Index; GS, Generation Scotland; PIS, Prescribing Information System.

Additional sociodemographic information collected in the questionnaire included gender, age, educational attainment, smoking status, and relationship status. Compared with the rest of GS, our sample was moderately older and contained more individuals with no school qualifications and also more degree-level educated individuals (Table 1, Fig. 1). Lifetime history of affective disorder (major depression and bipolar disorder) was obtained using the Structured Clinical Interview for DSM-IV Disorders [25]. Self-reported history of hypertension, heart disease, and diabetes was recorded. In addition, during the GS interview, a variety of cognitive tests were performed [24], including digit symbol from the Wechsler Adult Intelligence Scale III [27], logical memory from the Wechsler Memory Scale III [28], and verbal fluency [29]. From these tests, we derived a measure of general intelligence (*g*) as the first unrotated principal component, explaining 44% of the variance in scores [30], [31]. Psychological distress was measured using the General Health Questionnaire (GHQ)-28 (Likert scoring) [32].

**Table 1:** Sociodemographic, clinical, and cognitive characteristics of study populations compared with whole Generation Scotland cohort

Individual-level variables	GS:SFHS (*N* = 21,474)	Individuals in the present study (*N* = 10,244)	Subset of individuals in current study used in complete case multivariable logistic regression analysis (*N* = 9,043)
Female	12,674 (59.02%)	6,065 (59.21%)	5,329 (58.9%)
Age, 18–39 y	6,769 (31.52%)	3,072 (29.99%)^a^	2,797 (30.93%)^b^
Age, 40–64 y	12,346 (57.49%)	6,015 (58.72%)^a^	5,304 (58.65%)
Age, 65–99 y	2,359 (10.99%)	1,157 (11.29%)	942 (10.42%)
Affective disorder (SCID)	2,848 (13.26%)	1,329 (12.97%)	1,159 (12.82%)
Diabetes (self-report)	659 (3.07%)	323 (3.15%)	277 (3.06%)
Hypertension (self-report)	2,836 (13.21%)	1,297 (12.66%)^a^	1,125 (12.44%)
Cardiac disease (self-report)	777 (3.62%)	345 (3.37%)^a^	284 (3.14%)^b^
No school certificate	2,452 (11.42%)	1,432 (13.98%)^a^	1,296 (14.33%)^b^
Postgraduate education	6,323 (29.44%)	3,273 (31.95%)^a^	3,164 (34.99%)^b^
Smoker	3,662 (17.05%)	1,733 (16.92%)	1,484 (16.41%)^b^
Relationship status—single	6,720 (31.29%)	3,236 (31.59%)	2,866 (31.69%)^b^
GHQ Likert s	*16 (8.87)*	*15.73 (8.74)*^a^	*15.66 (8.69)*^b^
Wechsler Logical Memory Test I and II	*30.7 (8.48)*	*30.95 (8.15)*	*31.17 (8.05)*^b^
Mill Hill Vocabulary Test	*30.06 (4.76)*	*30.09 (4.66)*	*30.23 (4.62)*^b^
Wechsler Digit Symbol Substitution Task	*72.23 (17.22)*	*71.71 (17.15)*^a^	*72.52 (16.88)*^b^
Verbal Fluency Test	*39.71 (11.72)*	*39.89 (11.70)*^a^	*40.22 (11.65)*^b^

*Abbreviations*: GHQ, General Health Questionnaire; GS:SFHS, Generation Scotland: Scottish Family Health Study.

All values are totals with percentages, unless shown in italics where they are means with standard deviations in parentheses.

a Significant differences (alpha = 0.05) between Generation Scotland and Study Population as determined by Chi-square/t-tests.

b Significant differences (alpha = 0.05) between Study Population and subset used in multivariable logistic regression analysis as determined by Chi-square/t-tests.

### 2.4 Prescribing data and linkage

All Scottish citizens registered with a General Practitioner (GP; more than 96% of the population) are assigned a unique identifier (Community Health Index number). This was used to record link GS:SFHS questionnaire data to the national Prescribing Information System (PIS) administered by NHS Services Scotland Information Services Division [33]. PIS is a database of all Scottish NHS prescriptions for payments for medications prescribed by GPs, nurses, dentists, pharmacists, and hospitals where the medication was dispensed in the community. There is no prescription charge in Scotland. Hospital-dispensed prescriptions and over-the-counter medications are not included. Patient-level data have been available in PIS since April 2009 [34]. We obtained PIS-prescribing data for April 2009 to March 2011. We used the dates of dispensing, not prescription, when matching to self-report.

### 2.5 Matching prescribing to self-report

For each individual and medication type, concordance with GS:SFHS self-report was checked against PIS-prescribing record dispensing dates within a “fixed time window” [2], [4], [14], [16] including the month of questionnaire completion, and 2 months preceding (total 3 months), and also 5 months preceding (total 6 months). Most prescriptions, including in Scotland, are dispensed in quantities of 90 days duration or less [13], [35]. A previous Dutch study [12] also found that fixed time windows shorter than 90 days are less sensitive although the generalizability of this finding is uncertain. Accordingly, we used two fixed time windows, 3 and 6 months duration, to assess their relative benefits in terms of agreement, sensitivity, and positive predictive value (PPV).

To ensure all individuals had at least 6 months of potentially available prescribing records, we restricted analysis to GS:SFHS participants who had completed their medication questionnaire in September 2009 or later. This equated to 10,244 participants (6,065 females and 4,179 males) enrolled September 2009 to March 2011 (Table 1, Fig. 1). Of these, 96.5% had medication records available (the remainder were presumably not using prescribed medication) which compared with 95.6% for the whole GS cohort.

The PIS data allow medications to be identified by approved drug name and/or associated British National Formulary [36] paragraph code. Medication indication is not recorded. Our matching criterion for each medication type is detailed in Supplementary Table 4.

### 2.6 Missing data

The self-report questionnaire used a “Yes”/“No” checkbox, but some individuals ticked neither box (or data were otherwise missing, Table 2). In our main analysis, we treated each medication separately, excluding the missing self-report values for each case. However, to mitigate the potential of hereby introducing biases, or not accounting for individuals who intended to deny medication use by leaving the section blank, we conducted two additional analyses—one with all individuals with any missing data excluded (*n* = 7,836), and the other with missing data coded as denial of medication use (Supplementary Table 5).

**Table 2:** Medication self-report and prescribing data prevalence, agreements, sensitivities, specificities, and positive predictive values, measured on two fixed time windows—3 and 6 mo duration, respectively—in the present study (*n* = 10,244, including 6,065 females)

Medications	Total (*n*) completed question, with yes or no (%)	Medication prevalence according to self-report (%)	Medication prevalence according to PIS (%)^a^	3-mo fixed time window				6-mo fixed time window			
				Agreement κ (95% CI)	Sensitivity (95% CI)	Specificity (95% CI)	Positive predictive value (95% CI)	Agreement κ (95% CI)	Sensitivity (95% CI)	Specificity (95% CI)	Positive predictive value (95% CI)
Antidepressant^b^	8,333 (81.35)	9.60	10.10	0.84 (0.82–0.86)	0.90 (0.87–0.92)	0.99 (0.99–0.99)	0.90 (0.87–0.92)	0.85 (0.84–0.87)	0.85 (0.82–0.87)	0.99 (0.99–0.99)	0.89 (0.87–0.91)
Mood stabilizer^c^	7,977 (77.87)	1.17	1.32	0.40 (0.31–0.49)	0.41 (0.31–0.52)	0.99 (0.99–0.99)	0.41 (0.31–0.52)	0.42 (0.33–0.50)	0.40 (0.31–0.50)	0.99 (0.99–1.00)	0.45 (0.35–0.56)
Cholesterol-lowering medication	8,789 (85.80)	13.97	13.81	0.92 (0.91–0.94)	0.97 (0.96–0.98)	0.98 (0.98–0.99)	0.90 (0.88–0.92)	0.95 (0.94–0.96)	0.97 (0.95–0.97)	0.99 (0.99–0.99)	0.95 (0.94–0.97)
Antihypertensive	8,855 (86.44)	16.85	19.05	0.90 (0.89–0.91)	0.89 (0.87–0.91)	0.99 (0.99–0.99)	0.95 (0.94–0.96)	0.90 (0.89–0.91)	0.86 (0.85–0.88)	1.00 (0.99–1.00)	0.98 (0.97–0.98)
Aspirin	8,445 (82.44)	9.28	7.63	0.81 (0.78–0.83)	0.97 (0.95–0.98)	0.97 (0.97–0.98)	0.72 (0.68–0.75)	0.84 (0.82–0.86)	0.95 (0.93–0.96)	0.98 (0.97–0.98)	0.78 (0.75–0.81)
Insulin	8,016 (78.25)	1.11	0.97	0.87 (0.82–0.93)	1.00 (0.92–1.00)	1.00 (1.00–1.00)	0.78 (0.67–0.86)	0.93 (0.89–0.97)	1.00 (0.93–1.00)	1.00 (1.00–1.00)	0.88 (0.79–0.94)
HRT (female only)	4,794 (79.04)^a^	5.97	4.59	0.62 (0.57–0.68)	0.92 (0.87–0.96)	0.97 (0.96–0.97)	0.49 (0.43–0.55)	0.78 (0.74–0.82)	0.91 (0.86–0.94)	0.98 (0.98–0.98)	0.70 (0.64–0.75)
Oral contraceptives (female only)	4,849 (79.95)^a^	14.62	12.79	0.55 (0.51–0.59)	0.82 (0.78–0.86)	0.92 (0.91–0.92)	0.47 (0.43–0.51)	0.73 (0.70–0.76)	0.82 (0.79–0.85)	0.95 (0.95–0.96)	0.72 (0.68–0.75)

*Abbreviations*: CI, confidence interval; HRT, hormone replacement therapy; PIS, Prescribing Information System.

a Six-month time window used.

b Note that a broader definition of antidepressant than that shown in table, which included amitriptyline, returned an agreement of κ = 0.83 (0.81–0.85) at 6-mo time window with sensitivity of 0.75 (0.73–0.78).

c Note that a narrower definition of mood stabilizer than that shown in table, which comprised only lithium, sodium valproate, lamotrigine, and carbamazepine, returned an agreement of κ = 0.29 (0.20–0.38) at 6-mo time window with sensitivity of 0.21 (0.22–0.43).

### 2.7 Statistical analysis

All analyses were carried out using R version 3.2.3 [37]. Level of agreement between self-report and prescribing data was ascertained using Cohen's kappa (κ) method of rating interobserver variation [38]. Kappa scores of <0.40 were considered fair to poor; 0.41–0.60, moderate; 0.61–0.8, substantial; and >0.81, good or better [39], [40]. We also calculated sensitivity, specificity, and PPVs. Ninety-five percent confidence intervals (CIs) were included.

We performed multivariable logistic regression analysis on predictors of false negative self-report compared with true positive (sensitivity). Because of some covariate missing data, the sample size of this analysis was reduced to 9,043 for complete case analysis (Table 1, Fig. 1). Odds ratios (ORs) with 95% CI were calculated. Multiple testing was adjusted for using the False Discovery Rate method with significance level (alpha) 0.05. As GS is a partly family-based cohort, we adjusted for any correlation because of family relatedness using the Generalized Estimating Equations method [41].

## 3 Results

Of the 10,244 individuals in the study, 6,164 (60.17%) ticked “No” to every medication question (Fig. 1). In addition, 485 (4.74%) left blank or had missing data for every question. The proportion of completed responses differed between medications and was greatest for antihypertensives (86.44%) and lowest for mood stabilizers (77.87%, χ^2^ = 256.07, *P* < 2.2E-16; Table 2). The most commonly prescribed medication (6-month window) was antihypertensives, prevalence 19.05%, whereas antidepressants prevalence was 12.22% and mood stabilizers 1.32%. The prevalence of lifetime history of affective disorder in our sample was 12.66% (*n* = 1,297) for major depressive disorder and 0.31% for bipolar disorder (*n* = 32). The self-reported prevalence of hypertension was 12.66% (*n* = 1,297), heart disease 3.37% (*n* = 345), and diabetes 3.15% (*n* = 323; Table 1).

### 3.1 Agreement and validity

**Fig. 2:**
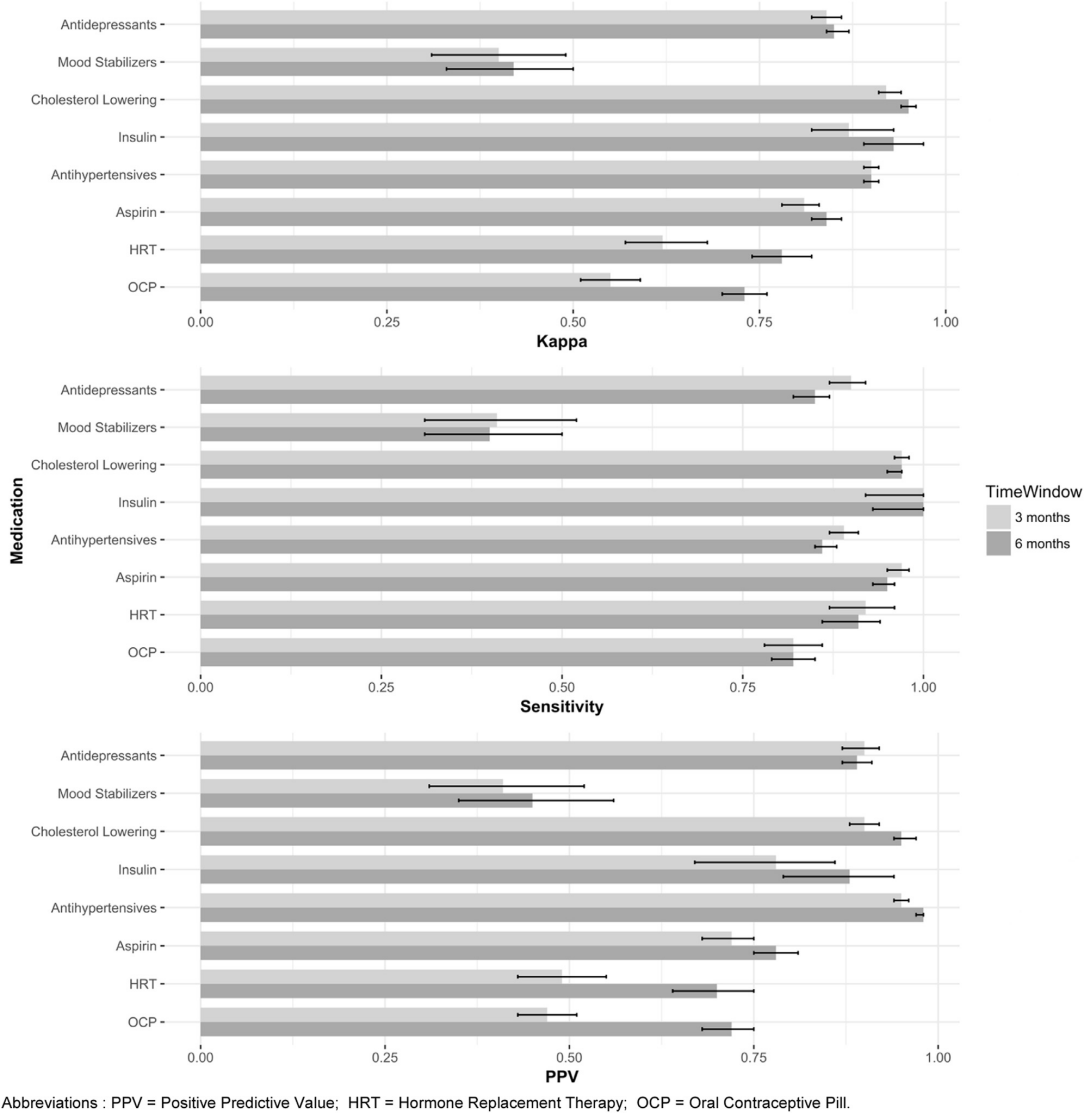
Agreement and validity of medication self-report compared with prescribing data as gold standard. Using 3- and 6-month fixed time windows, with 95% confidence intervals. HRT, hormone replacement therapy; OCP, oral contraceptive pill; PPV, positive predictive value.

Agreement (Table 2, Fig. 2) between medication self-report and prescribing data was generally very good across medication classes. Greatest agreement was found for cholesterol-lowering medication (κ = 0.95, CI 0.94–0.96; 6-month fixed time window unless otherwise stated). Agreement for antidepressants (κ = 0.85, CI 0.84–0.87) was lower than antihypertensives (κ = 0.90, CI 0.89–0.91), but still within the highest kappa banding of >0.81. By contrast, agreement for mood stabilizers was moderate-poor (κ = 0.42, CI 0.33–0.50). Comparing the 6-month fixed time window to 3-month, κ scores were higher although only to a degree beyond 95% CIs in the case of HRT and oral contraceptives.

Self-report sensitivity (Table 2, Fig. 2) was slightly reduced in the 6-month time window vs. 3-month, but was still >0.80 for all medications except mood stabilizers. Antidepressant sensitivity (0.85, CI 0.82–0.87) was comparable to antihypertensives (0.86, CI 0.85–0.88). Sensitivity for mood stabilizers was comparatively poor (0.40, CI 0.31–0.50) indicating a high rate of false negatives.

The PPV (Table 2, Fig. 2) for antidepressant use (0.89, CI 0.87–0.91) was substantial, albeit less than antihypertensives and cholesterol-lowering drugs, and contrasted with modest PPV for mood stabilizers (0.45, CI 0.35–0.56). The 6-month fixed time window significantly improved PPV for most medication groups, with the greatest effect for HRT and oral contraceptives (which nevertheless showed relatively moderate PPV in both time windows).

### 3.2 Predictors of failure to self-report medication usage

Multivariable logistic regression (Table 3) found no covariates universally associated, across all medications, with failure to self-report medication usage, as determined by the prescribing data gold standard. General intelligence (*g*) was not associated with increased false negatives for any medication. Psychological distress (GHQ) reduced odds of false negatives for antidepressants (OR 0.98, CI 0.96–1.00, *P*_FDR_ = 0.081) and mood stabilizers (OR 0.96, CI 0.91–1.01, *P*_FDR_ = 0.197), but this relationship was not significant for multiple testing.

**Table 3:** Odds ratios (with 95% confidence intervals) for factors associated with failure to self-report medication use (false negatives) as determined by prescribing data as gold standard

Individual-level variables	Antidepressants	Mood stabilizes	Cholesterol-lowering medication	Antihypertensives	Aspirin	Oral contraceptives (females only)
Female sex	0.67 (0.42–1.09)	0.75 (0.24–2.33)	1.62 (0.80–3.30)	**1.75 (1.16–2.62)**	1.14 (0.52–2.48)	–
Age	**0.97 (0.95–0.99)**	0.96 (0.91–1.02)	0.95 (0.92–0.99)	**0.94 (0.92–0.96)**	*0.94 (0.90*–*0.99)*	1.01 (0.98–1.04)
Affective disorder	*0.55 (0.35*–*0.87)*	**0.09 (0.02–0.35)**	0.72 (0.22–2.42)	0.82 (0.47–1.44)	0.70 (0.19–2.51)	1.31 (0.69–2.49)
Diabetes	–	–	0.42 (0.13–1.40)	**0.30 (0.13–0.70)**	–	–
Hypertension	–	–	*0.28 (0.11*–*0.71)*	**0.04 (0.02–0.06)**	0.49 (0.23–1.06)	–
Heart disease	–	–	0.30 (0.07–1.25)	0.82 (0.45–1.50)	*0.15 (0.03*–*0.65)*	–
No school certificate	0.60 (0.26–1.32)	**17.0 (2.3–125.84)**	0.45 (0.12–1.72)	0.66 (0.37–1.17)	0.88 (0.28–2.82)	0.65 (0.07–5.89)
Higher education	1.17 (0.70–2.00)	1.27 (0.25–6.35)	1.63 (0.65–1.09)	0.85 (0.54–1.34)	1.27 (0.44–3.64)	1.41 (0.80–2.49)
Smoker	0.90 (0.52–1.54)	0.12 (0.02–0.082)	1.30 (0.45–3.76)	*1.84 (1.09*–*3.11)*	1.58 (0.59–4.21)	*1.98 (1.13*–*3.46)*
Ex-smoker	0.66 (0.38–1.11)	0.44 (0.10–2.00)	1.32 (0.59–2.92)	1.40 (0.93–2.12)	0.71 (0.28–1.81)	1.18 (0.65–2.14)
Relationship status—couple	0.89 (0.56–1.41)	2.03 (0.59–7.01)	1.31 (0.58–2.97)	0.96 (0.63–1.47)	0.91 (0.40–2.08)	0.78 (0.48–1.28)
General intelligence (g)	0.85 (0.70–1.04)	0.76 (0.46–1.26)	0.85 (0.65–1.11)	1.02 (0.85–1.21)	1.17 (0.83–1.66)	0.92 (0.74–1.15)
Psychological distress (GHQ Likert)	*0.98 (0.96*–*1.00)*	0.96 (0.91–1.01)	0.99 (0.95–1.04)	0.99 (0.97–1.01)	1.00 (0.95–1.04)	1.02 (0.99–1.04)

*Abbreviation*: GHQ, General Health Questionnaire.

Significant associations are shown in bold (alpha = 0.05 and adjusted for multiple testing by False Discovery Rate method) and near-significant associations (alpha <0.10) are shown in italics.

The following factors were used as controls and do not appear in the table: male sex; age 18 to 39 years; secondary school education only; no affective disorder found on SCID; no history of self-reported high blood pressure/heart disease/diabetes; smoking status—never smoked; relationship status—single.

Insulin and hormone replacement therapy (HRT) are not shown in the table as no significant associations with predictors were found.

There was reduced discordant self-reporting for several medications if the patient had a history of an illness for which that medication was indicated, such as affective disorder and mood stabilizers (OR 0.09, CI 0.02–0.35, *P*_FDR_ = 0.005), and hypertension and antihypertensives (OR 0.04, CI 0.02–0.06, *P*_FDR_ <0.001). Similar associations were found for affective disorder and antidepressants and cardiac disease and aspirin, with *P* values of <0.1 after correcting for multiple testing.

Age and gender showed no consistent association although older age was associated with lower false negatives for antihypertensives, antidepressants, and possibly aspirin (*P*_FDR_ = 0.074), and female gender was associated with increased false negatives for antihypertensives (OR 1.75, CI 1.16–2.62, *P*_FDR_ = 0.020).

### 3.3 Influence of missing data

Recoding missing data as negative self-report (Supplementary Table 5) resulted in somewhat lower levels of agreement and lower sensitivities for all medications. However, agreement remained good for antidepressants (κ = 0.81, CI 0.79–0.83) and poor for mood stabilizers (0.34, CI 0.26–0.41). There was a demonstrable reduction in sensitivity for antidepressants (0.78, CI 0.75–0.80), but this reduction was not confined to psychiatric medications, being found also in antihypertensives (0.79, CI 0.77–0.81).

## 4 Discussion

In this population-based cohort, we found substantial to very good agreement between medication self-report and electronic prescribing records, for most medications studied. We hypothesized that psychiatric medications would show less agreement and systematic under-reporting. Agreement for mood stabilizers was indeed considerably worse although we found evidence of both under- and over-reporting (false positives). However, for antidepressants, the agreement, sensitivity, and PPV were broadly comparable to other medications studied. We did not identify any generalizable single predictors of failure to self-report prescribed medications, for psychiatric medications, or for medications generally. However, past medical history of an indicated health condition showed the strongest effect in promoting self-report accuracy across classes, and this was also true for psychiatric medications.

In general, the 6-month fixed time window outperformed the 3-month for agreement and PPV, at the expense of modest loss of sensitivity. This was most evident for HRT and oral contraceptives in women, which could imply these medications are dispensed in longer time cycles, and require longer fixed time windows relative to other medications.

### 4.1 Predictors of discordant self-report

We found that a medical history of an indicated health condition for a given medication, such as affective disorder for mood stabilizers or hypertension for antihypertensives, reduced the odds of false negatives. If systematic under-reporting of psychiatric medications due to self-stigma was taking place, we might have expected to find the reverse. Relationship status and educational status did not predict discordance, except in the case of mood stabilizers where lack of school qualifications was associated with false negative reporting. This could indicate reduced understanding of the definition of “mood stabilizer” among the less educated, but might also represent association between lesser educational achievement and use of medications (such as antipsychotics) included in our definition of mood stabilizers.

We found that general intelligence (*g*) did not influence concordance of medication self-report with prescribing data, which to our knowledge has not been previously reported. We also believe we are the first to investigate psychological distress and medication self-report. Interestingly, although psychological distress might be posited as a potential factor in under-reporting psychiatric medications (e.g., through self-stigma), we found some evidence of a relationship between the increased GHQ score and greater sensitivity of self-reporting of antidepressants (*P* < 0.1). Gender was not generally associated with accuracy, except in the case of antihypertensives, where increased odds of false negatives (OR 1.75, CI 1.16–2.62) were found, perhaps indicating greater usage of these medication types for non-antihypertensive purposes among females.

### 4.2 Questionnaire phrasing

One possible explanation for the poor agreement, sensitivity, and PPV for mood stabilizers is confusion among questionnaire respondents about the meaning of “mood stabilizer.” There is no consensus definition of mood stabilizer among clinicians [42], and laypersons may therefore be unsure as to its meaning. Klungel [8] has previously reported that sensitivity of medication self-report is influenced by the specificity of question phrasing. In our matching to prescribing data, we used a broad definition of mood stabilizers, but when a narrower definition (excluding antipsychotics) was used, the agreement was even worse (κ = 0.29, CI 0.20–0.38).

### 4.3 Comparison with other studies

Supplementary Table 6 describes the agreement of this present study, using the 6-month fixed time window, with other large published studies. We report a higher level of agreement (κ = 0.86) for antidepressants than Nielsen (κ = 0.66) [4], Rauma (κ = 0.65) [2], and Noize (κ = 0.81) [20]. When making comparisons with studies performed in other healthcare systems, it is important to recognize the variations between countries in prescribing legislation and access to medication. Scotland has a national health system, with no prescription charges, and prescribing data is collated nationally, which might explain a higher concordance with self-report and prescribing data that might be possible in some comparator studies.

Kwon [10] compared survey antidepressant self-report in a longitudinal depression study (*n* = 164) with pharmacy claims data and a 3-month fixed window and found substantial levels of agreement (κ = 0.69). Interestingly, where there were discrepancies in prescription record antidepressant use, they found on notes review that most cases could be explained by antidepressants being used for other indications or due to recent discontinuation. In our study, we attempted to minimize the rate of antidepressant false positives because of other indications by excluding amitriptyline from our searches (amitriptyline is widely prescribed but now rarely for depression in the United Kingdom).

With regard to mood stabilizers, a recent study comparing self-reported medication use in a genetic study of schizophrenia (*n* = 905) [16] found substantial levels of agreement (κ = 0.74) between self-report of mood stabilizers and an administrative prescription database. This is a much higher level of agreement than found in our study although we note that Haukka's was not a community-based sample and had a much higher prevalence of mood stabilizer used. A comparison of a postal medication survey (*n* = 11,031) with national prescription records reported by Rauma [2] found substantial levels of agreement for antidepressant reporting (κ = 0.65) but poor agreement (κ = 0.30) for other psychoactive medications, a result more comparable with our own findings.

### 4.4 Study strengths and weaknesses

Our study used a large (*n* = 10,244) population-based cohort linked to high-fidelity Scottish PIS records (capture rate in excess of 95%) [34]. Self-report was via a short, simply worded questionnaire which obviated interviewer bias and did not require long-term recall of medication use. Response rate was high. We used a variety of methods to compare the two data sources over two fixed time windows and performed covariate analysis of predictors of discordant self-report.

However, our method of verifying medication utilization took no account of dose and concordance with medication was assumed. Patients may be prescribed a drug but not fill their prescription (primary noncompliance) although our use of date of dispensing rather than prescribing date would have obviated this to an extent, it would still be unknown if the dispensed drug was collected. In addition, patients may not take the drug or not take as intended (secondary noncompliance), and concordance can be as low as 50% for antidepressants and antihypertensives [4], [43]. In addition, the questionnaire referred to “regularly” taken medication whereas our method recorded any prescription within the fixed time window as positive use. The absence of data in PIS on medication indication increased the risk of overinclusion and false positives, particularly for medications with broader indications although we attempted to decrease this using our exclusion criteria (Supplementary Table 4). Fixed time windows also potentially record false positives for medications discontinued during the window, but before self-report, although this is more common with medications taken acutely, such as antibiotics [12].

We must, therefore, concede that prescription data is by its nature an imperfect gold standard although its use enables very large sample sizes which improve overall accuracy. The use of prescribing data as a gold standard involves some strong assumptions, including that the patient could not have obtained the medication without it being recorded in the prescribing data. The extent to which this is true depends on a variety of variables, including the medication type, prescribing legislation of the country of study, and the movement of individual patients between healthcare providers. Indeed, some studies are performed on the basis of self-report as gold standard to analyze the validity of clinical or prescribing records [44]. However, the advantage of prescribing data as a gold standard is that it is an objective measure, with definitions of medication usage that can be readily replicated across studies and countries (whereas self-report questionnaires can vary considerably in definition and interpretation); which can be utilized at large scale across multiple medication types; and which is not subject to potential recall and desirability biases of self-report studies [45].

Data linkage is also a fast-moving field, and although the PIS data from 2011 we used in this study had high fidelity and a capture in excess of 95%, future studies using larger datasets and more complex linkage may enable even more accurate estimates of validity. For example, as data linkage improves, cross-referencing to other sources of clinical data such as GP and hospital records should assist identifying true cases and also reduce the incidence of false positives for those who have discontinued medication through the time windows analyzed.

As discussed, the use of the term “mood stabilizer” may have caused confusion. Many individuals did not tick either checkbox, and response rate differed between medication types, from 86.44% for antihypertensives to 77.87% for mood stabilizers. This may have reflected variations in understanding of, or willingness to answer, the question and could have biased our results or inflated the kappa scores. However, we demonstrated that recoding these missing data as denial of use still produced substantial levels of agreement (Supplementary Table 5). The Cohen's kappa method itself may inflate values depending on the proportion of subjects in each category [46]; hence, we have also tabulated the raw proportions (Supplementary Table 7). GS:SFHS is a partly family-based cohort, and this could potentially have introduced some correlation bias into our analysis although we accounted for this in our multivariable regression through Generalized Estimating Equations.

## 5 Conclusion

Our study provides convincing evidence that medication self-report is accurate compared with prescribing data, particularly for medication classes that are more precisely definable. We have shown that self-report of antidepressant use meets the highest threshold for Cohen's kappa agreement and can be considered valid for research and clinical purposes. Our analysis of potential patient-level predictors of reporting discordance, such as gender, age, education, and general intelligence, did not identify generalizable factors across all medication classes although there was some evidence that medical history of an indicated condition improves sensitivity of self-report. As discussed previously, medication-level factors such as range of possible indications, and length of dispensing cycles, may also be important when validating self-report across a fixed time window with prescribing data as gold standard.

Our study also demonstrates the utility of record linkage of longitudinal population-based cohorts to nationally administered prescribing datasets, as a useful adjunct to epidemiologic and large biobanking studies. Using administrative health data for verification and quality control of self-report has applications beyond epidemiologic studies and can be potentially exploited in clinical applications, such as data-linked clinical support tools acting as adjuncts to clinical interview, and in formulating predictive models of disease risk [47].
