# Supplementary material for: Self-reported medication use validated through record linkage to national prescribing data
Source: J Clin Epidemiol. 2018 Feb;94:132–42. doi: 10.1016/j.jclinepi.2017.10.013 (PMC5808931; doi:10.1016/j.jclinepi.2017.10.013)
Supplement: Supplementary Tables 4–7 [file mmc1.docx]

**Validation of Self-Reported Medication Use Through Record Linkage To National Prescribing Data**

**Supplementary Material**

**Table 4:** Matching and exclusion criteria used for prescribing database searches.

| **Self-Reported Medication** | **Matching criteria used in PIS** | **Exclusion criteria** |
| --- | --- | --- |
| **Cholesterol lowering medication (e.g. simvastatin)** | BNF Paragraph code “212000”. |  |
| **Antihypertensives** | BNF Paragraph codes “205051” (ACE inhibitors); “205052” (Angiotension II antagonists); “204000” (beta blockers); “206020” (calcium channel blockers); “202010” (thiazides and aldosterone antagonists); “202020” (loop diuretics); “202030” (potassium sparing diuretics); “205040” (alpha adrenoceptor blocking drugs); “202040” (combined K sparing diuretics); “205010” (vasodilator antihypertensive drugs); “205020” (centrally acting hypertensives); “205053” (renin inhibitors) | Records containing : “amiloride hydrochloride”, “bumetanide”, “eplerenone”, “sotalol hydrochloride”, “amiloride hydrochloride with bumetanide”, “co-amilofruse”, “triamterene with furosemide”, “sildenafil”, “clonidine hydrochloride” [These medications are not specifically indicated for hypertension] |
| **Insulin** | BNF Paragraph codes “601011” and “601012” |  |
| **Hormone replacement therapy** | BNF Paragraph codes “604011” |  |
| **Oral contraceptive pill or mini pill** | BNF Paragraph codes containing “^7030*” | BNF Paragraph codes “703050” (emergency contraceptives); “703040” (spermicidals); “703030” (contraceptive devices) |
| **Aspirin** | Prescribable Item Approved Name containing “aspirin” | Formulations containing aspirin as additional ingredient |
| **Antidepressants** | BNF Paragraph codes containing “^4030*”: “403010” (tricyclic antidepressants), “403020”(monoamine oxidase inhibitors), “403030”(selective serotonin reuptake inhibitors) and “403040”(other antidepressant drugs). | Records containing: “amitriptyline” [An antidepressant which is no longer commonly prescribed for depression in the UK and which is often used short-term for other indications] |
| **Mood stabilizers** | Prescribable Item Approved Name containing : “lithium”; “carbamazepine”; “lamotrigine”; “valproate”; “amisulpride”; “olanzapine”; “aripiprazole”; “risperidone”; “quetiapine” |  |

| Table 5.  Comparison of Agreement (Cohen’s kappa), Sensitivity and Positive Predictive Value with prescribing data for study population with (A) missing self-report medication data recoded as medication denied (B) all records with any missing self-report medication data excluded | | | | | | |
| --- | --- | --- | --- | --- | --- | --- |
|  | **A.** All missing data recoded as medication denied, Six-month fixed time window  (N=10,244) | | | **B.** Complete cases analysis with all missing data of medication responses excluded, Six month fixed time window  (N=7836) | | |
|  | Agreement  κ | Sensitivity | Positive Predictive Value | Agreement  κ | Sensitivity | Positive Predictive Value |
|  |  |  |  |  |  |  |
| Antidepressants | **0.81**  (0.79-0.83) | **0.78**  (0.75-0.80) | **0.89**  (0.87-0.91) | **0.78**  (0.75-0.81) | **0.73**  (0.69-0.77) | **0.86**  (0.82-0.89) |
| Mood stabilizers | **0.34**  (0.26-0.41) | **0.28**  (0.21-0.36) | **0.45**  (0.35-0.56) | **0.29**  (0.19-0.40) | **0.24**  (0.15-0.35) | **0.39**  (0.25-0.54) |
| Cholesterol lowering medication | **0.94**  (0.93-0.95) | **0.93**  (0.92-0.95) | **0.95**  (0.94-0.97) | **0.93**  (0.92-0.95) | **0.92**  (0.89-0.94) | **0.96**  (0.93-0.97) |
| Antihypertensives | **0.85**  (0.84-0.86) | **0.79**  (0.77-0.81) | **0.98**  (0.97-0.98) | **0.82**  (0.80-0.84) | **0.74**  (0.70-0.77) | **0.97**  (0.96-0.98) |
| Insulin | **0.91**  (0.87-0.96) | **0.95**  (0.88-0.99) | **0.88**  (0.79-0.94) | **0.92**  (0.86-0.98) | **1.00**  (0.86-1.00) | **0.86**  (0.72-0.95) |
| HRT (female only) | **0.76**  (0.72-0.80) | **0.85**  (0.80-0.90) | **0.70**  (0.64-0.75) | **0.76**  (0.70-0.82) | **0.84**  (0.76-0.90) | **0.71**  (0.62-0.78) |
| Contraceptive (female only) | **0.72**  (0.69-0.75) | **0.79**  (0.76-0.82) | **0.72**  (0.68-0.75) | **0.71**  (0.67-0.74) | **0.77**  (0.73-0.81) | **0.71**  (0.67-0.75) |
| Aspirin | **0.82**  (0.80-0.85) | **0.90**  (0.88-0.93) | **0.78**  (0.75-0.81) | **0.80**  (0.76-0.84) | **0.88**  (0.83-0.91) | **0.77**  (0.70-0.80) |

| Table 6. Comparison of Study Results With Other Published Studies Of Similar Methodology (Fixed Time Window). | | | | | |
| --- | --- | --- | --- | --- | --- |
| Study and method | **Medication** | **Kappa (95% CI)** | **Sensitivity** | **Specificity** | **PPV** |
| Current Study 6 Month Fixed Time Window | **Antidepressants** | **0.85** (0.84-0.87) | **0.85** (0.82-0.87) | **0.99** (0.99-0.99) | **0.89** (0.87-0.91) |
| Nielsen et al (2008) (n=16,688) Interview based Danish nationally representative survey compared with national prescription records. Age 16+. 90-day time window (and legend time duration – not shown). | Antidepressants | 0.66 (0.62-0.70) |  |  |  |
| Caskie et al (2006) (n=1430) Longitudinal USA population based study ages 23-97 years. Comparison of medication “brown bag” interview with pharmacy prescription records (4 fixed month time window). | Antidepressants |  | 0.86 |  |  |
| Rauma et al (2013) (n=11031) Postal questionnaire of postmenopausal Finnish women (age 58-67, mean age 62.3) compared to national prescription register, 4 month fixed time window (also 12 month fixed time window – not shown) | Antidepressants | 0.65 | 0.55 | 0.99 |  |
| Haapea et al (2010) (n=7625) Postal questionnaire of Finnish birth cohort (all born 1966, data collected 1997) compared with register of social insurance institution, 6-month fixed time window | Antidepressants | 0.68 (0.61-0.76) |  |  |  |
| Richardson et al (2013) (n=2621) Irish Longitudinal Study on ageing. 50 years’ age and older community dwelling population compared with pharmacy dispensing records, 6-month fixed time window | Psychanaleptics | 0.69 (0.65-0.73) |  |  |  |
| Noize et al (2009) (n=4112) French older adult (65 years+) cohort study comparing questionnaire to national health insurance system, 60-day fixed time window (also 30 day – not shown) | Antidepressants | 0.81 (0.77-0.84) | 83 | 98.2 | 81.9 |
| Haukka et al (2007) (n=905) Finnish population based genetic study of schizophrenia of which 422 had schizophrenia or bipolar disorder. Age range 30-65. Participants were interviewed about their medication and compared to social insurance prescription database. 180-day fixed time window. | Antidepressants | 0.77 |  |  |  |
|  |  |  |  |  |  |
| Current Study 6 Month Fixed Time Window | **Mood stabilizers** | **0.42** (0.33-0.5) | **0.40** (0.31-0.50) | **0.99** (0.99-1.00) | **0.45** (0.35-0.56) |
| Haukka et al (2007) | Mood stabilizers  Lithium | 0.74  0.96 |  |  |  |
| Nielsen et al (2008) | Antipsychotics | 0.73 (0.68-0.78) |  |  |  |
| Rauma et al (2013) | Other psychoactive medication | 0.30 | 0.29 | 0.97 |  |
| Haapea et al (2010) | Antipsychotics | 0.77 (0.69-0.85) |  |  |  |
| Noize et al (2009) | Antipsychotics | 0.76 (0.66-0.84) | 69.9 | 99.8 | 83.8 |
| Richardson et al (2013) | Psycholeptics | 0.59 (0.55-0.63) |  |  |  |
|  |  |  |  |  |  |
| Current Study 6 Month Fixed Time Window | **Cholesterol lowering medication** | **0.95** (0.94-0.96) | **0.97** (0.95-0.97) | **0.99** (0.99-0.99) | **0.95** (0.94-0.97) |
| Monster et al (2002) (n=8592) Questionnaire from Netherlands population based study (ages 28-75 years, mean 49.5) compared with pharmacy data 1 year fixed time window | Lipid lowering drugs | 0.81 | 0.85 |  | 0.79 |
| Richardson et al (2013) | Lipid modifying agents | 0.73 (0.69-0.77) |  |  |  |
| Noize et al (2009) | Lipid lowering agents | 0.85 (0.84-0.87) | 0.86 | 0.98 | 0.95 |
|  |  |  |  |  |  |
| Current Study 6 Month Fixed Time Window | **Antihypertensives** | **0.90** (0.89-0.91) | **0.86** (0.85-0.88) | **1.00** (0.99-1.00) | **0.98** (0.97-0.98) |
| Nielsen et al (2008) | Cardiovascular system | 0.80 (0.78-0.81) |  |  |  |
| Caskie et al (2006) | Beta blockers, Ca channel blockers, ACE inhibitors, diuretics |  | 0.89 – 0.95 |  |  |
| Monster et al (2002) | Antihypertensives | 0.69 | 0.89 |  | 0.62 |
| Haapea et al (2010) | Beta blocking agents | 0.55 (0.46-0.64) |  |  |  |
| Richardson et al (2013) | Beta blocking agents, Calcium channel blockers, Diuretics | 0.77 (0.73-0.81) -  0.80 (0.76-0.84) |  |  |  |
| Noize et al (2009) | Antihypertensives | 0.86 (0.84-0.87) | 90.3 | 96.3 | 96.8 |
| Sjahid et al (1998) (n=1682) Dutch cohort study of older adults (age 55+), patient interview compared with pharmacy records, 6 month fixed time window | Beta blocking agents, Calcium channel blockers, Diuretics | 0.90-0.97 |  |  |  |
| Rauma et al (2013) (n=11031) Postal questionnaire of postmenopausal Finnish women (age 58-67, mean age 62.3) compared to national prescription register, 4 month fixed time window (also 12 month fixed time window – not shown) | Diuretics | 0.82 | 0.83 | 0.98 |  |
|  |  |  |  |  |  |
| Current Study 6 Month Fixed Time Window | **Aspirin** | **0.84** (0.82-0.86) | **0.95** (0.93-0.96) | **0.98** (0.97-0.98) | **0.78** (0.75-0.81) |
| Nielsen et al (2008) | Antithrombotic agents | 0.75 (0.70-0.80) |  |  |  |
| Caskie et al (2006) | Salicylates |  | 0.40 |  |  |
| Richardson et al (2013) | Antithrombotic agents | 0.72 (0.68-0.76) |  |  |  |
|  |  |  |  |  |  |
| Current Study 6 Month Fixed Time Window | **Insulin** | **0.93** (0.89-0.97) | **1.00** (0.93-1.00) | **1.00** (1.00-1.00) | **0.88** (0.79-0.94) |
| Nielsen et al (2008) | Insulins and analogues | 0.82 (0.77-0.87) |  |  |  |
| Caskie et al (2006) | Diabetic agents |  | 0.97 |  |  |
| Haapea et al (2010) | Antidiabetics | 0.92 (0.87-0.97) |  |  |  |
| Richardson et al (2013) | Drugs used in diabetes | 0.86(0.82-0.89) |  |  |  |
| Noize et al (2009) | Drugs used in diabetes | 0.93 (0.91-0.95) | 0.91 | 0.99 | 0.96 |
|  |  |  |  |  |  |
| Current Study 6 Month Fixed Time Window | **Hormone replacement therapy** | **0.78** (0.74-0.82) | **0.91** (0.86-0.94) | **0.98** (0.98-0.98) | **0.70** (0.64-0.75) |
| Nielsen et al (2008) | Hormone replacement therapy | 0.51(0.47-0.55) |  |  |  |
| Caskie et al (2006) | Oestrogens |  | 0.82 |  |  |
| Monster et al (2002) | Hormone replacement therapy | 0.49 | 0.60 |  | 0.46 |
| Lokkegard et al (2004) Questionnaire to Danish nurses (n=2666) compared to administrative national health service prescribing databases (time window up to 9 years, non-fixed) | Hormone replacement therapy |  | 0.74 (0.72-0.78) | 0.98 (0.97-0.99) |  |
|  |  |  |  |  |  |
| Current Study 6 Month Fixed Time Window | **Oral contraceptives** | **0.73** (0.70-0.76) | **0.82** (0.79-0.85) | **0.95** (0.95-0.96) | **0.72** (0.68-0.75) |
| Monster et al (2002) | Oral contraceptives | 0.65 | 0.80 |  | 0.64 |

**Table 7: Self-Reported Medication Utilization Compared To Prescribing (PIS) Records (Six Month Fixed Time Window)**

|  | Self Report  Negative  Prescribing  Negative  True  Negatives | % | Self Report  Negative  Prescribing  Positive  False  Negatives | % | Self Report  Positive  Prescribing  Negative  False  Positives | % | Self Report  Positive  Prescribing  Positive  True  Positives | % | Total (excluding NAs) | Blank/ Missing Data | Blank/  Missing Data and Prescribing  Positive | %  Blank/ Missing and  Prescribing  Positive |
| --- | --- | --- | --- | --- | --- | --- | --- | --- | --- | --- | --- | --- |
| Antidepressants | 7404 | 88.85 | 129 | 1.55 | 87 | 1.04 | 713 | 8.56 | 8333 | 1911 | 78 | 4.08% |
| Mood stabilizers | 7821 | 98.04 | 63 | 0.79 | 51 | 0.64 | 42 | 0.53 | 7977 | 2267 | 46 | 2.03% |
| Cholesterol lowering medication | 7519 | 85.55 | 42 | 0.48 | 56 | 0.64 | 1172 | 13.33 | 8789 | 1455 | 40 | 2.75% |
| Antihypertensives | 7134 | 80.56 | 229 | 2.59 | 34 | 0.38 | 1458 | 16.47 | 8855 | 1389 | 159 | 11.44% |
| Aspirin | 7626 | 90.30 | 35 | 0.41 | 175 | 2.07 | 609 | 7.21 | 8445 | 1799 | 29 | 1.61% |
| Insulin | 7927 | 98.89 | 0 | 0.00 | 11 | 0.14 | 78 | 0.97 | 8016 | 2228 | 4 | 0.18% |
| HRT | 4488 | 93.62 | 20 | 0.42 | 86 | 1.79 | 200 | 4.17 | 4794 | 1271 | 14 | 1.105 |
| OCP | 4029 | 83.09 | 111 | 2.29 | 200 | 4.12 | 509 | 10.50 | 4848 | 1216 | 24 | 1.97% |
